# Method of Detecting the Presence of a Minute Quantity of Bile in the Animal Fluid

**Published:** 1846-04

**Authors:** 


2. *Method for detecting the presence of a minute quantity of Bile in the Animal Fluids.*—The ordinary method pursued for the purpose of detecting the colouring matter of bile (biliphæin of Simon, *choleptyrrhin* of Berzelius) in fluids supposed to contain it, consists in adding a portion of nitric acid to the suspected fluid, when, if bile be present, there is immediately produced a beautiful green colour, which by degrees becomes changed successively to blue, violet, red and yellow. It is essential, however, that the quantity of bile should be considerable, in order that these various changes of colour should take place on the addition of nitric acid; if the quantity is but small, there is only produced a greenish colour, which shortly becomes changed to yellow without passing through the degrees of blue and red, for the production of which the quantity of bile present is not sufficient. Dr. Heller,* moreover, observes, that he has frequently known bile to exist in urine and other fluids without its presence being indicated, or any change of colour effected by nitric acid in the ordinary way of applying this test. He states, however, that if any fluid in which bile exists, contains a portion of albumen, the nitric acid, by coagulating the albumen, will detect the smallest possible quantity of bile, for the coagulum assumes at once either a bluish, or perfectly blue, or greenish colour, and if the bile exists in large quantity the coagulated albumen will accordingly assume a green, and then a reddish colour. In pursuing this mode of testing for the presence of bile, Heller recommends that to the suspected fluid, say urine, a considerable excess of strong nitric acid is to be added, and should there be produced by this means none of the ordinary colours indicative of the presence of bile, then that to another portion of the fluid some albumen dissolved in water (serum of blood, if

*Archiv. fur Physiol. und Pathol. Chemie and Microsc., Heft I.

at hand) is to be added, and well mixed; a little nitric acid is now to be poured into the mixture, which, after being stirred up, is to be left at rest for the albuminous precipitate to form: if bile be present, this precipitate of coagulated albumen presents a bluish or greenish-blue colour, but if it be not, then the coagulum is simply white (though, after a time, it assumes a yellowish tint, owing to the action of the nitric acid, but this is quite independent of the presence of bile). Thus, therefore, the simplest plan to detect bile in a non-albuminous fluid consists in making the fluid albuminous, and then treating it with nitric acid; should blood be the fluid requiring to be tested, nitric acid may be added at once to the serum, which contains albumen in abundance.

The microscope is capable of still further improving upon this mode of procedure, and of rendering this test applicable in cases where the suspected fluid is too small in quantity to be examined satisfactorily in an ordinary test-tube; for this purpose, Donné* recommends that a drop of a suspected fluid be placed between two slips of glass, and a little nitric acid added whilst the object is beneath the microscope; immediately upon the acid coming in contact with the fluid characteristic colours are struck, should bile be present. In this way Donné was enabled to determine that an abscess communicated with the intestine, by simply examining a drop of the pus discharged.—*Lond. Med. Gaz.*, Oct., 1845., in *Amer. Jour. of Med. Sciences.*

* Cours de Microscopie, page 212.

